# Supplementary figures and images for: The Spanish Osteopathic Practitioners Estimates and RAtes (OPERA) study: A cross-sectional survey
Source: PLoS One. 2020 Jun 15;15(6):e0234713. doi: 10.1371/journal.pone.0234713 (PMC7295231; doi:10.1371/journal.pone.0234713)

***Fig 1****. Distribution (%) of participants across the different Spanish regions*


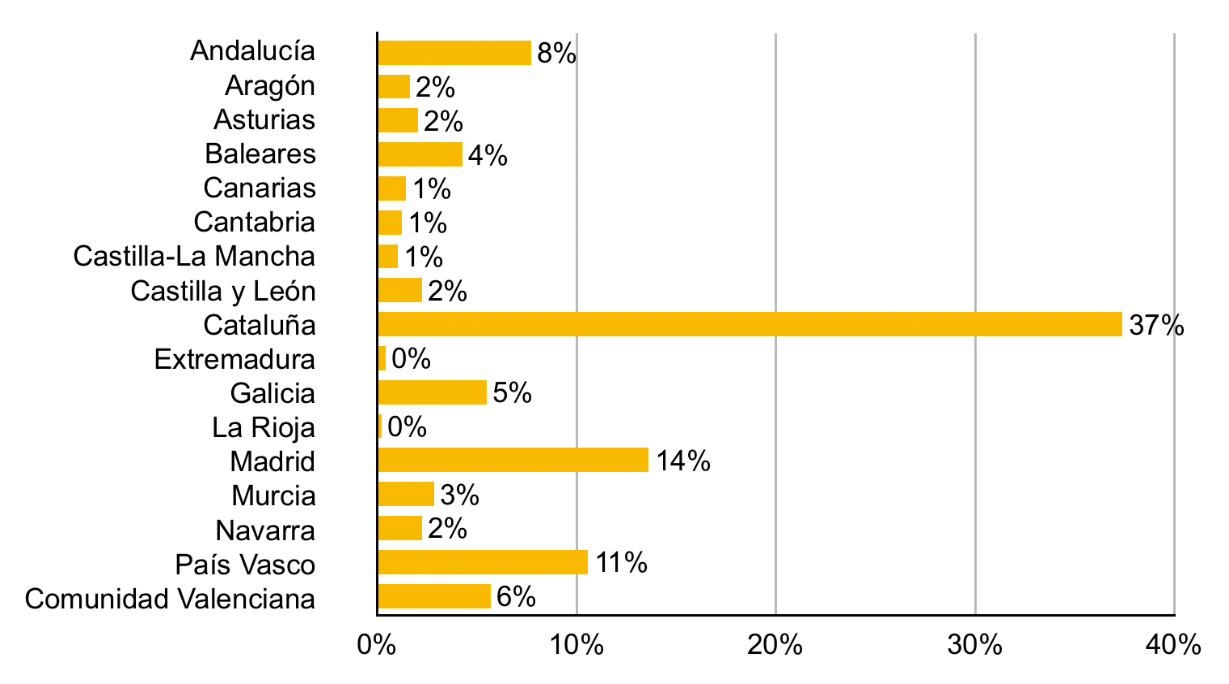

Supplement: S1 Fig — (DOCX) [file pone.0234713.s001.docx]
