# Supplementary material for: The Spanish Osteopathic Practitioners Estimates and RAtes (OPERA) study: A cross-sectional survey
Source: PLoS One. 2020 Jun 15;15(6):e0234713. doi: 10.1371/journal.pone.0234713 (PMC7295231; doi:10.1371/journal.pone.0234713)
Supplement: S1 Table — (DOCX) [file pone.0234713.s002.docx]

| **Table 1:** Relation age and gender among respondents | | |
| --- | --- | --- |
|  | Male (%) | Female (%) |
| 20-29 | 4.2 | 5.6 |
| 30-39 | 29.5 | 23.7 |
| 40-49 | 22.2 | 9.2 |
| 50-59 | 2.9 | 0.7 |
| 60-65 | 0.5 | 0.5 |
| >65 | 0.3 | 0.0 |
