# Supplementary material for: The Spanish Osteopathic Practitioners Estimates and RAtes (OPERA) study: A cross-sectional survey
Source: PLoS One. 2020 Jun 15;15(6):e0234713. doi: 10.1371/journal.pone.0234713 (PMC7295231; doi:10.1371/journal.pone.0234713)
Supplement: S2 Table — (DOCX) [file pone.0234713.s003.docx]

| **Table 2:** Osteopathic Associations and Registers (alphabetic order) | | | |
| --- | --- | --- | --- |
|  | N | Total registered osteopaths | % |
| AOC | 17 | 265 | 6.4 |
| AETCB | 0 | 265 | 0.0 |
| ANAOST | 1 | 265 | 0.3 |
| APREO | 20 | 265 | 7.5 |
| APTN_COFENAT | 12 | 265 | 4.5 |
| ASEPROST | 0 | 265 | 0.0 |
| Asociación de osteópatas de Catalunya | 1 | 265 | 0.3 |
| Asociación de Osteopatía Sacrocraneal | 0 | 265 | 0.0 |
| Asociación española de osteopatía infantil | 0 | 265 | 0.0 |
| ROE | 134 | 265 | 50.5 |
| ROFE | 44 | 265 | 16.6 |
| ROP | 8 | 265 | 3.0 |
| SEMMO | 0 | 265 | 0.0 |
| SEMO | 16 | 265 | 6.0 |
| SEMOOYM | 0 | 265 | 0.0 |
| TENACAT | 1 | 265 | 0.3 |
| Osteópatas sin Fronteras | 3 | 265 | 1.1 |
| APROETENA | 8 | 265 | 3.0 |
