# Supplementary material for: The Spanish Osteopathic Practitioners Estimates and RAtes (OPERA) study: A cross-sectional survey
Source: PLoS One. 2020 Jun 15;15(6):e0234713. doi: 10.1371/journal.pone.0234713 (PMC7295231; doi:10.1371/journal.pone.0234713)
Supplement: S3 Table — (DOCX) [file pone.0234713.s004.docx]

| **Table 3:** Other professional activities (n=517) |  |  |
| --- | --- | --- |
|  | N | % |
| Clinical physical therapist | 170 | 32.8 |
| Teaching classes in osteopathy | 109 | 21.0 |
| Other professional activities | 69 | 13.3 |
| Other clinical health related profession | 44 | 8.5 |
| Supervising osteopathic clinical practices | 42 | 8.1 |
| Research | 26 | 5.0 |
| Supervising thesis in osteopathy | 23 | 4.4 |
| Professional society or osteopathic association | 16 | 3.0 |
| Clinical physician | 8 | 1.5 |
| Other activities | 34 | 6.5 |
