# Supplementary material for: The Spanish Osteopathic Practitioners Estimates and RAtes (OPERA) study: A cross-sectional survey
Source: PLoS One. 2020 Jun 15;15(6):e0234713. doi: 10.1371/journal.pone.0234713 (PMC7295231; doi:10.1371/journal.pone.0234713)
Supplement: S4 Table — (DOCX) [file pone.0234713.s005.docx]

| **Table 4:** Academic degrees |  |  |  |
| --- | --- | --- | --- |
|  | N | Total participants | % |
| physical therapist | 391 | 517 | 75.6 |
| massage therapist | 126 | 517 | 24.3 |
| other health related | 53 | 517 | 10.2 |
| sport scientists | 43 | 517 | 8.3 |
| other not health related | 41 | 517 | 7.9 |
| chiropractor | 24 | 517 | 4.6 |
| nurse | 19 | 517 | 3.6 |
| medical doctor | 12 | 517 | 2.3 |
| none  midwife | 9  1 | 517  517 | 1.7  0.1 |
