# Supplementary material for: The Spanish Osteopathic Practitioners Estimates and RAtes (OPERA) study: A cross-sectional survey
Source: PLoS One. 2020 Jun 15;15(6):e0234713. doi: 10.1371/journal.pone.0234713 (PMC7295231; doi:10.1371/journal.pone.0234713)
Supplement: S5 Table — (DOCX) [file pone.0234713.s006.docx]

| **Table 5:** Patients reason for consultation | | | | | |
| --- | --- | --- | --- | --- | --- |
| % | never | seldom | regularly | often | always |
| chronic problem | 0.0 | 0.9 | 7.3 | 36.9 | 54.7 |
| acute problem | 0.0 | 1.7 | 9.2 | 35.9 | 53.0 |
| prevention | 1.1 | 8.7 | 25.1 | 35.9 | 29.0 |
| post surgery | 5.0 | 30.7 | 41.7 | 15.6 | 6.7 |

Numbers in table are %
