# Supplementary material for: The Spanish Osteopathic Practitioners Estimates and RAtes (OPERA) study: A cross-sectional survey
Source: PLoS One. 2020 Jun 15;15(6):e0234713. doi: 10.1371/journal.pone.0234713 (PMC7295231; doi:10.1371/journal.pone.0234713)
Supplement: S6 Table — (DOCX) [file pone.0234713.s007.docx]

**Table 6:** Reasons for referring patients

| % | Not important | Rather not important | Rather important | Important | Very important |
| --- | --- | --- | --- | --- | --- |
| not my speciality | 4.4 | 2.5 | 6.0 | 20.7 | 66.3 |
| indication of undiagnosed pathology | 0.5 | 1.1 | 8.5 | 24.9 | 64.8 |
| worsening primary symptoms | 0.7 | 3.4 | 11.2 | 26.3 | 58.2 |
| worsening pre-existing symptoms | 1.1 | 5.0 | 12.7 | 32.6 | 48.3 |
| review of actual medication | 1.3 | 3.4 | 19.9 | 33.8 | 41.3 |
| insufficient progression of the patient | 0.5 | 1.1 | 14.3 | 36.3 | 47.5 |
| dissatisfaction patient | 4.8 | 7.1 | 24.1 | 31.1 | 32.6 |
| increase anxiety/depression patient | 1.9 | 5.4 | 21.8 | 39.0 | 31.7 |
| other | 17.0 | 6.9 | 24.7 | 21.4 | 29.7 |

Numbers in table are %
