# Supplementary material for: The Spanish Osteopathic Practitioners Estimates and RAtes (OPERA) study: A cross-sectional survey
Source: PLoS One. 2020 Jun 15;15(6):e0234713. doi: 10.1371/journal.pone.0234713 (PMC7295231; doi:10.1371/journal.pone.0234713)
Supplement: S7 Table — (DOCX) [file pone.0234713.s008.docx]

**Table 7:** Consultation policy

| % | No | Yes | % No | Yes |
| --- | --- | --- | --- | --- |
| Inform about the confidentiality policy | 103 | 414 | 19.9 | 80.0 |
| Inform about data protection policy | 113 | 404 | 21.8 | 78.1 |
| Inform about accompanied visit policy for minors | 126 | 391 | 24.3 | 75.6 |
| Inform about accompanied visit policy for intimate zone treatment | 136 | 381 | 26.3 | 73.6 |
| Inform about the consultation cancellation policy | 315 | 202 | 60.9 | 39.0 |

Numbers in table are %
