# Supplementary material for: The Spanish Osteopathic Practitioners Estimates and RAtes (OPERA) study: A cross-sectional survey
Source: PLoS One. 2020 Jun 15;15(6):e0234713. doi: 10.1371/journal.pone.0234713 (PMC7295231; doi:10.1371/journal.pone.0234713)
Supplement: S8 Table — (DOCX) [file pone.0234713.s009.docx]

**Table 8:** Content of osteopathic training

|  | No | Yes | % No | | % Yes |  |
| --- | --- | --- | --- | --- | --- | --- |
| clinical reasoning | 22 | 495 | 4.2 | | 95.7 |  |
| identification of red alerts | 22 | 495 | 4.2 | | 95.7 |  |
| interpretation of medical images | 52 | 465 | 10.0 | | 89.9 |  |
| documentation of the clinical history | 81 | 436 | 15.6 | | 84.3 |  |
| indications for ordering medical images | 90 | 427 | 17.4 | 82.5 | | |
| methodology and examination | 100 | 417 | 19.3 | | 80.6 |  |
| working in clinical osteopathy practice | 113 | 404 | 21.8 | | 78.1 |  |
| professional ethics and regulations | 117 | 400 | 22.6 | | 77.3 |  |
| clinical hygiene | 158 | 359 | 30.5 | | 69.4 |  |
| communication in osteopathy practice | 175 | 342 | 33.8 | | 66.1 |  |
| diagnostic laboratory results | 218 | 299 | 42.1 | | 57.8 |  |
| biomedical statistics | 228 | 289 | 44.1 | | 55.9 |  |
| clinical pharmacology | 264 | 253 | 51.0 | | 48.9 |  |
| first aid and basic life support | 277 | 240 | 53.5 | | 46.4 |  |
| psychology of pain and disability | 300 | 217 | 58.0 | | 41.9 |  |

Numbers in table are %
