# Supplementary material for: The Spanish Osteopathic Practitioners Estimates and RAtes (OPERA) study: A cross-sectional survey
Source: PLoS One. 2020 Jun 15;15(6):e0234713. doi: 10.1371/journal.pone.0234713 (PMC7295231; doi:10.1371/journal.pone.0234713)
Supplement: S9 Table — (DOCX) [file pone.0234713.s010.docx]

**Table 9:** Osteopathic identity

| % | strongly disagree | | | | disagree | neutral | agree | strongly agree | |
| --- | --- | --- | --- | --- | --- | --- | --- | --- | --- |
| I’m proud to be an osteopath | 0.5 | | | | 1.7 | 2.3 | 16.8 | 78.5 | |
| Being a health practitioner is important to me | | | | 2.9 | 1.7 | 5.6 | 14.3 | 75.4 | |
| Being osteopath is important to me | | 0.5 | | | 1.5 | 3.0 | 21.0 | 73.6 | |
| Define myself as a health practitioner | | | 4.2 | | 2.7 | 8.3 | 15.4 | | 69.2 |
| Define myself as an osteopath | | 3.8 | | | 6.1 | 12.3 | 28.0 | | 49.5 |

Numbers in table are %
