# Supplementary material for: The Spanish Osteopathic Practitioners Estimates and RAtes (OPERA) study: A cross-sectional survey
Source: PLoS One. 2020 Jun 15;15(6):e0234713. doi: 10.1371/journal.pone.0234713 (PMC7295231; doi:10.1371/journal.pone.0234713)
Supplement: Attachment — Submitted filename: Response to Reviewers.pdf [file pone.0234713.s011.pdf]

“The Spanish Osteopathic Practitioners Estimates and RAtes (OPERA) study: A cross sectional survey”
Response to peer review: Manuscript number PONE-D-20-08905

Reviewer #1

Comment	Answer:
1. This study contributes to the data needed on osteopaths within different countries. Meanwhile participation rate was low, recruitment process was difficult with no official registry.<br>2. The main comment that I would transmit is that authors wanted to show all the data collected in the manuscript. Moreover, there are a lot of tables and figures (9 tables and 5 figures). I would suggest authors to think about reducing the data shown, in order to help readers to focus on the most important data. Some of the data should be transferred in the supplemental material. For example, the figure about the regions where respondents practiced does not bring many information to the readers, as it is not representative of the prevalence of osteopaths in each region of Spain.	1. We would like to thank the reviewer’s comments and his/her help to improve our manuscript. Certainly, we faced several difficulties in the recruitment phase of the study. Due to the unregulated and heterogeneous osteopathic scenario in Spain it is difficult to conduct studies on a national scale. However, we believe that it is precisely in these countries that these surveys could help to get a better picture of the situation and thus to define strategies to improve the profession. In fact, our limited capacity to show representative national data ultimately demonstrate the need for a unique and central census. Concerning the participation rate, it is within the expected range and superior to the values needed to show differences between categories according to Barlett et al(1). Hence, the responder rate is large enough for the purpose of the survey. This reference has been added to the manuscript in the limitations section where shortcomings related to sample size and response rate are discussed. Finally, we would like to emphasize that, regardless of the final number, this study is the largest observational study carried out in Spain regarding osteopath’s prevalence and practice characteristics, and at least is a first step towards evaluating further aspects of the profession.<br><br>(1) Bertlett J.E. , Kotrlik J.W. and Higgins C.C. Organizational research: determining appropriate sample size in survey research (2001). Information technology, learning, and performance journal. 19 (1); 43-50<br><br>2. We’ve transferred to the supplemental material 2 tables and the figure suggested by the reviewer

Comment	
**Abstract :**<br><br>1. In the methods, I would suggest to have more elements described in the methods of the abstract: questionnaire already used in other studies, translated into Spanish,… (elements of what is written in the survey tool part of the manuscript).<br>2. It seems that there is no registry of all osteopaths within Spain. For this reason, the authors reached the potential participants through different ways. This should be clarified shortly in abstract. This should be also clarified in the manuscript. For example, it would be interesting to know how many osteopaths were reached by emails (even if a part of participants were not reached by emails).	**Amendments:**<br>1. More questionnaire details have been included in the methods section of the abstract.<br>2. Clarifications have been included in the abstract and also in the manuscript. Unfortunately, it is impossible to know exactly how many osteopaths were finally reached because the registers and associations acted as intermediaries. The research team requested the registers to transmit the information to their associates and provide them with information concerning the survey on the OPERA website. We know the final number of participants and within this group the number of participants for each register. However, we do not know the response rate within each register because we do not know the total number of members in each of the organisations. In summary, we cannot know how many accessed the survey thanks to the registers, or thanks to our social media campaign.

"The Spanish Osteopathic Practitioners Estimates and RAtes (OPERA) study: A cross sectional survey"
Response to peer review: Manuscript number PONE-D-20-08905

Comment	
**Introduction:**<br><br>1. The questionnaire should be better described: was the questionnaire changed since the first version (I think it was van Dun et al. State of affairs of osteopathy in the Benelux: Benelux Osteosurvey 2013)? If yes, what was changed?<br>2. The authors mentioned that "A secondary objective is to compare the results with other similar studies to establish potential differences across countries. » I am not sure that it can be an objective, as the authors did not analyze the data from other countries. They compared their results with other countries, which is not an objective, but one part of the discussion.	**Amendments:**<br>1. Details on the adaptation process have been added in the Methods section.<br>2. The sentence "A secondary objective is to compare the results with other similar studies to establish potential differences across countries" has been removed

Comment	**Amendments:**
**Methods:**<br><br><u>Survey tool:</u> it is mentioned that the questionnaire was adapted to "new insights and regional requirements". I would recommend that the authors describe more precisely what was changed in the questionnaire.	Details on the adaptation process have been added:<br>*"Adaptation were due to the influence of a more extensive and international research team in the project and the involvement of the project-leader (PvD) in a related professional doctorate project (1). The main adaptations introduced in the Spanish version, after having analyzed the Benelux Osteosurvey, were the omission of some original questions that did not seem to contribute essential information and the omission/adaptation and others with questioned validity. Questions related to potentially sensitive information (e.g. fees) were adapted to closed-ended questions with categories. Some other open-ended questions were adapted to closed-ended after the implementation of the Benelux Osteosurvey results. Finally, a new chapter with two questions about "professional identity and views as an osteopath" was added.*<br><br>*(1) Dornieden, R. (2019) Exploration of the characteristics of German osteopaths and osteopathic physicians - survey development and implementation. Prof. Doctorate thesis. University of Bedfordshire.*

Comments	
**Methods:**<br><br><u>Statistical analysis:</u><br>1. If my understanding is right, diploma in osteopathy are delivered by private schools. I do not understand why inclusion criteria was not based on the practitioners who had a diploma, but on practitioners who considered themselves as osteopaths. I suggest to explain better the reason for this decision.<br>2. "Statistical analyses were based on a univariate and multivariate approach. R statistical programme (v. 3.1.3) was used." I do not find any univariate or multivariate data. It is not clear why this sentence was mentioned. On the other hand, it would be probably interesting to know if there are some differences linked with sex and age of osteopaths (having another professional activity, number of patients each week, …).	**Amendments:**<br><br>1. Yes, the reviewer is right. However, what is considered a "diploma in osteopathy" in Spain is difficult to define due to the high variety of training programmes available. A large number of Osteopathic Education Institutes (OEI) is active in Spain, offering a wide range of titles, ranging from short courses to long training courses. In this cross-sectional study, we wanted to capture all possible realities, despite the type of training, and we defined our inclusion criteria accordingly. A clarification has been included in the manuscript.<br>2. The whole paragraph has been modified. In fact, we have revised all the issues related to statistics, especially to clarify the terms "prevalence" and "representative sample". We did not attempt to analyse differences according to specific variables because we cannot be sure that we have a representative sample of the osteopaths in Spain and we cannot correct for imbalances. Therefore, any inference would be at risk of being incorrect.

“The Spanish Osteopathic Practitioners Estimates and RAtes (OPERA) study: A cross sectional survey”
Response to peer review: Manuscript number PONE-D-20-08905

Comment	**Amendments:**
**Results:**<br><br>1. Tables: I suggest to write only one number after the comma.<br>2. Table 2: I do not think that this table is useful. I would suggest to remove it.<br>3. Figure 1. I do not think that this figure is useful, as it represents the respondents and not the number of osteopaths for each region. I would suggest to remove it.<br>4. Supporting information S1: promotion videos is a very good idea. Meanwhile, I am not sure that it will useful to give the access to readers in the manuscript.<br>5. Professional identity: I find this part very important and interesting. I would suggest to have a figure with some of these results. On the other hand, I would recommend to authors decide if data in figure 3, 4 and 5 should really be in a figure.<br>6. Table 5. I would suggest to reduce the number of groups for “number of patients on consultation each week”. It is hard to read now. Same for fee.	1. This has been changed in the whole manuscript, tables and supplementary files have been created.<br>2. Table 2 has been moved to the supplementary files.<br>3. Figure 1 has been moved to the supplementary files.<br>4. Supporting information S1 (with videos has been removed).<br>5. In a first version of the manuscript, the information related to professional identity was also included as a Table. However, we felt that this was somehow a repetition of the text and in order to reduce the number of tables, we finally decided to keep only the text which allowed to display the most relevant information. At the same time, in order to reduce the length of text and given the complexity of the data we think that the information related to Fig 3,4 and 5 (now 2,3 and 4) are adequately presented in this format.<br>6. We have collapsed some categories of Table 5 (now table 3) as also suggested by reviewer 2.

“The Spanish Osteopathic Practitioners Estimates and RAtes (OPERA) study: A cross sectional survey”
Response to peer review: Manuscript number PONE-D-20-08905

Comment	
**Discussion:**<br><br>1. “Although respondents come from almost all Spanish regions, the higher concentration of practitioners is located in Catalunya and Madrid (figure 1), which are the autonomous communities with the largest population in Spain.” As this result could be linked with the recruitment process, I would suggest to remove this sentence.<br>2. It would be interesting if you also compare your results with data from Switzerland: https://journals.plos.org/plosone/article?id=10.1371/journal.pone.0224098<br>3. I would suggest, where possible, to reduce the length of the discussion	**Amendments:**<br>1. The sentence has been removed.<br>2. Data and comparisons with the reference provided have been included.<br>3. The discussion has been reduced in length and also modified according to reviewer’s 2 suggestions.

Comment	
**Conclusion:**<br><br>1. The conclusion should be better written, only linked with what the data says. For example, I am not sure that the study estimated the prevalence of osteopaths. It also did not use representative data. Moreover, it does not give reliable information on where osteopathy is practiced.<br>2. “Finally, the information provided can be used by stakeholders and policymakers in a potential future regulation of the profession in Spain.” I would remove this sentence. Other studies are needed to get sufficient reliable data.	**Amendments:**<br>1. The whole conclusion has been rewritten, avoiding mentions to “prevalence”, “representative data” and “reliable”.<br>2. We agree with the reviewer that other studies are needed to get sufficient reliable data. However, we think that the data presented in this study, could be considered valuable for stakeholders and policymakers and give them a more reliable view of the profession. We have adapted the sentence to better reflect our idea.

“The Spanish Osteopathic Practitioners Estimates and RAtes (OPERA) study: A cross sectional survey”
Response to peer review: Manuscript number PONE-D-20-08905

Reviewer #2

Comment	
Thank you for the opportunity to review this manuscript. I provide some comments that I hope the authorship team will find useful.<br><br>Overall, this manuscript could benefit from another close copyedit to strengthen the written English/grammar. Some examples are provided below, but a full review is needed.	We would like to thank reviewer 2 for the comments provided and his/her help on improving our manuscript. We have amended the majority of them and clarified some aspects that certainly needed attention. A second English/grammar revision has been made by the team with also contributions of a professional translator.

Comment	
**Background:**<br><br>1. P6: I think the term "heterogenous" would suit better than "inhomogenous"<br>2. P6: Also, do you mean training and education methods? rather than processes?<br>3. P7: Please avoid paragraphs of only two sentences. The two paragraphs on this page (starting "In 2013..." and "Within this uncertain...") can be edited to form one more cohesive paragraph.<br>4. P7: edit - "is still unregulated as is the case in Spain"<br>5. P8: edit - "numerous training programs has resulted in the coexistence of..."<br>6. P8: in what way does the unpublished studies provide unreliable information?	**Amendments:**<br>1. Amended<br>2. Amended<br>3. Amended<br>4. Edited<br>5. Edited<br>6. Sentence changed “despite some **academic dissertations** addressing this issue[13–15], there is only one **published study** providing this type of information[7].”

Comment	
**Materials and methods:**<br><br>1. Please replace all numbers less than 10 with the words (e.g. ten) where they occur in sentences.<br>2. P10: in the abstract you refer to the prevalence of Osteopaths in Spain as part of your aim but it is not listed in the main methods section. I don't think you can confer prevalence from your methodology anyway.<br>3. P12: how was the survey adapted? what was the process you undertook to do this?	**Amendments:**<br>1. Amended.<br>2. The word “prevalence” has been removed from the abstract and the rest of the manuscript.<br>3. Details on the adaptation process have been added:<br>*“Adaptation were due to the influence of a more extensive and international research team in the project and the involvement of the project-leader (PvD) in a related professional doctorate project (1). The main adaptations introduced in the Spanish version, after having analyzed the Benelux Osteosurvey, were the omission of some original questions that did not seem to contribute essential information and the omission/adaptation of questions with questioned validity. Questions related to potentially sensitive information (e.g. fees) were adapted to closed-ended questions with categories. Some other open-ended questions were adapted to closed-ended after the implementation of the Benelux Osteosurvey results. Finally, a new chapter with two questions about "professional identity and views as an osteopath" was added.*<br><br>*(1) Dornieden, R. (2019) Exploration of the characteristics of German osteopaths and osteopathic physicians - survey development and implementation. Prof. Doctorate thesis. University of Bedfordshire.*

“The Spanish Osteopathic Practitioners Estimates and RAtes (OPERA) study: A cross sectional survey”
Response to peer review: Manuscript number PONE-D-20-08905

Comment	Amendments:
**Results (part 1):**<br><br>1. Unless it is a journal requirement, I don't think you need to provide any more than one decimal place for most of your data. It adds nothing of value to the paper and distracts from the findings in the tables and text.<br>2. Although this is a style preference, you could try reworking some of your sentences so that you are not starting so many with a numeric value. This will reduce the overall manuscript length as you will then be able to convert quite a few words to numbers.<br>3. P19: I am not sure I agree that "specific pathologies" and "gynecological conditions" count as a population group? Could this be reworded?<br>4. Table 3: The last three percentages have a comma "," instead of a decimal "."<br>5. P21: Based on your data, does this mean some self-identifying as osteopaths may have no osteopathic qualifications at all? How would they have been trained? I understand the challenges of an unregulated environment, but this warrants some close attention in the discussion (I think).<br>6. Table 4: Even though the frequency is higher, I recommend moving "other activities" to the end of the list in this table.	1. Has been changed in the whole manuscript, tables and supplementary files.<br>2. We have changed 7 sentences to avoid starting with a numerical value.<br>3. We are referring to patients’ groups with specific conditions. We’ve added “patients with specific pathologies” and “patients with gynecological conditions”. It relates to osteopaths who frequently work with patients having certain conditions/diseases outside of the “most common” complaints seen by osteopaths (e.g. patients with Parkinson disease, children with cerebral palsy, men with chronic prostatitis). In Spain, there are many specific courses and CPDs focused on gynecological complaints and therefore, this area is seen as a specialty and even some osteopaths advertise their expertise on this specific subject and treat mainly this type of patients (e.g. chronic pelvic pain, sexual dysfunctions, dysmenorrhea)<br>4. Has been changed. Thank you.<br>5. All respondents had an osteopathic training but not all of them (2%) had a prior academic (university) degree as it is not mandatory in Spain. We’ve included this clarification (“a prior”) to avoid misunderstandings.<br>6. Amended. The table has been moved to supplementary files according to reviewers 1 suggestions.

Comment	Amendments:
**Results (part 2):**<br><br>7. P22: It is unsurprising that such a high number "strongly define as an osteopath" given those who don't would not have likely responded to the survey. More interesting is the 15% who completed the survey but do NOT strongly identify as an osteopath.<br>8. P22: The percentage of osteopaths agreeing that osteopathy should be regulated as a paramedical profession is repeated. Once in the sentence and once in parentheses at the end.<br>9. Table 5: The value of so many categories of patient numbers is not apparent. Could you collapse down some categories? Same for the number of new consultations per week. There are such lower numbers in 16-20 and >20 categories that they could easily be combined. And again for the fee categories.<br>10. P28: The sentence beginning "Increase in level of primary symptoms" does not need to be separate. It can be included in the previous paragraph.	7. We understand the reviewer's comment but the question is very relevant in the Spanish context where the osteopath with a prior education in physiotherapy is the most common profile. Some of them, although being osteopaths, are more prone to self-identifying with their physiotherapy profile. Moreover, there is a strong political and academical controversy in Spain regarding a possible regulation of osteopathy. In this struggle, a number of osteopaths with a prior education in physiotherapy claim that osteopathy is a speciality of physiotherapy. In fact, they oppose the recognition of osteopathy as a separate profession. Although the majority of the respondents are in favour of a regulation, there are a number who still identify themselves as physiotherapists and who want to link osteopathy to the physiotherapy profession. We think that those osteopaths represent the 15% in our survey.<br>8. Removed. Thank you.<br>9. We have collapsed some categories of Table 5 (now table 3) as also suggested by reviewer 1.<br>10. Amended. Thank you.

“The Spanish Osteopathic Practitioners Estimates and RAtes (OPERA) study: A cross sectional survey”
Response to peer review: Manuscript number PONE-D-20-08905

Comment
**Discussion:**<br>My most substantial comment for this manuscript relates to the discussion. It feels unecessarily long. I think that the authors could identify 3 to 5 (maximum) of the most important discussion points and provide a nuanced engagement with those topics. As it stands, the manuscript overviews numerous findings and compares them superficially to existing research without providing the reader with any in-depth consideration of the context, meaning and application of the results. One such example is the possible feminisation of the workforce. More discussion about why this may be occuring would be great. As another example, the point about the alignment between osteo and physio could be paralleled with the osteo/chiropractic links in Australia. The finding about low frequency of cranial/visceral is also interesting. Is that a result of a shift in evidence priorities? Training and curriculum? Ultimately the discussion would be strengthened by providing a deeper engagement with the implications of the findings rather than simply comparing them with other studies.
**Answer:**
Thank you for the comment. We’ve tried to adapt our discussion according to the reviewer’s suggestions. Please note that, as in essence our study has a descriptive purpose, the application of the results is not always straightforward. In addition, we think that the comparisons with other countries are appropriate as it allows the reader to capture the main differences and could trigger adaptations within the community to further develop the profession taking other countries as an example. However, we have reinforced the interpretation of our results by adding reflections in the four sections that constitute our discussion. Please find below the structure of this revised version and the main amendments made:<br><br>⇒ **1st paragraph:** Brief summary of our results and first comparison with similar surveys. A specific research has been done on the possible causes that explain the feminisation of the profession. Since no data has been found we’ve reinforced this idea adding some references showing also this shift in health care disciplines in general.<br>⇒ **Osteopathic training:** One more reference has been included as suggested by reviewer 1. The variety of training programs is explained by the lack of European regulation prior to the existence of a couple of official documents.<br>⇒ **Practice characteristics and professional fees:** Some information has been removed to reduce the length. Comparisons with other surveys are still there. However, the discussion highlights important aspects as interprofessional care and referrals and we provide three possible causes that could explain why a more fluid interdisciplinary relationship is lacking within the Spanish scenario. In the

final part we discuss the fees, the specific features of private healthcare system in Spain and its impact on osteopathic services. We argue that an integration of osteopathy in the Spanish healthcare system would benefit patient’s health.

⇒ **Patients, diagnostic and therapeutic modalities:** Again, some information has been removed to reduce the length. However, relevant new data, recently published, has been included as well as a mention that links the Spanish with the Australian situation related to dual qualifications. This section finalizes with a reflection on the Spanish context and providing a clear picture on the gap between the legal situation and the osteopath’s desire to become a regulated profession.

Comment	
Conclusions:<br><br>Once the discussion has been revised, the conclusion will also need attention as it currently makes claims that are not substantiated within the study such as "offers compelling data in order to better assess better the inclusion of osteopathic services within the Spanish healthcare system". For a claim like this to be upheld your discussion will need to engage with this point directly. Also, I am not sure you can use the phrase "nationally representative" as you don't have a basis for comparison. You can say it draws on a national sample, though.	**Amendments:**<br><br>The sentence has been removed, we have omitted the word “representative” and the strengths of our claims have been balanced according to our study design, results and limitations.
